# Light People: Professor Chennupati Jagadish

**DOI:** 10.1038/s41377-021-00533-6

**Published:** 2021-06-01

**Authors:** Hui Wang

**Affiliations:** Department of International Cooperation, Changchun Institute of Optics, Fine Mechanics and Physics, Chinese Academy of Sciences, 3888 Dong Nan Hu Road, 130033 Changchun, China

**Keywords:** Quantum dots, Semiconductor lasers

## Abstract

In 2018, the Indian film “Starting Line” focused the public’s attention on the issue of education in India. It depicted the length some Indian parents were willing to go to secure educational resources for their children, as well as the difficulties faced by those disadvantaged in society in their fight for equal educational opportunities. In reality, many brilliant young Indian talents have been able to study in Australia through a fund set up by Prof. Chennupati Jagadish, a Distinguished Professor of the Australian National University. Prof. Jagadish is a Fellow of the Australian Academy of Science and the Australian Academy of Technological Sciences and Engineering. In 2018 he was awarded a UNESCO Prize for his contribution to the development of nanoscience and nanotechnology. He holds many positions, and has won numerous awards. What started Prof. Jagadish on his scientific research career? How did he become the respected scientist he is today? What was his intention in setting up the educational fund for students from developing countries? What advice does he have for young researchers? Here are the answers from Prof. Jagadish.

## Abstract (web summary)

Prof. Jagadish, awarded a UNESCO Prize for his contribution to the development of nanoscience and nanotechnology, holding many positions and numerous awards, talks exclusively to Light.

## Graphical abstract

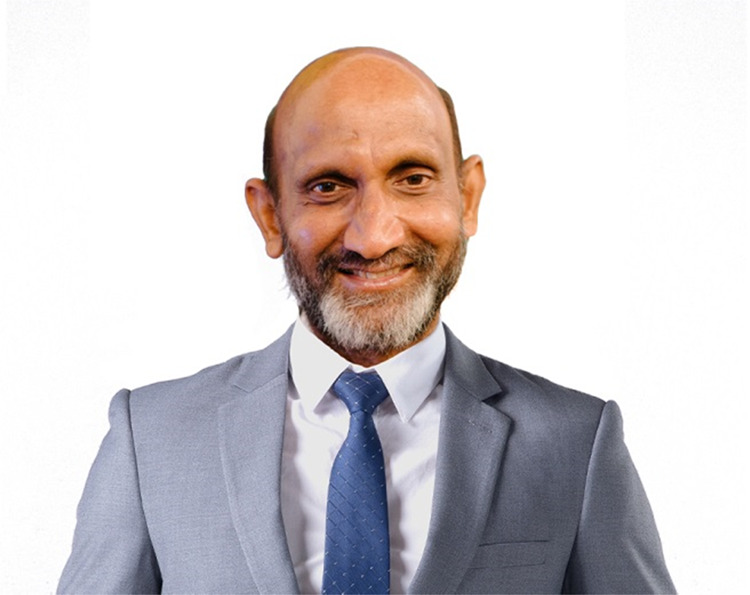



**Biography:** Prof. Jagadish is a Distinguished Professor of the Australian National University. He is serving as Editor-in-Chief (EIC) of Applied Physics Reviews and also served as EIC of Progress in Quantum Electronics. His research interests include compound semiconductor optoelectronics, nanotechnology, photovoltaics and neurotechnology. He is a Fellow of the Australian Academy of Science and the Australian Academy of Technological Sciences and Engineering, a Fellow of IEEE, APS, OSA, SPIE, AAAS, TWAS, etc., a Foreign Member of the US National Academy of Engineering, a Foreign Fellow of the Indian National Science Academy and Indian National Academy of Engineering.

He has been awarded the Australian Federation Fellowship (2004–2009) and the Australian Laureate Fellowship (2009–2014) by the Australian Research Council. He has received many awards, including The Quantum Device Award (ISCS, 2010), IEEE Pioneer in Nanotechnology Award (2015), IEEE Photonics Society Engineering Achievement Award (2015), Distinguished Fellow of the Chinese Academy of Sciences President’s International Fellowship Initiative (2016), Welker Award from ISCS (2017) and Lyle Medal from the Australian Academy of Science(2019).

**1. You have cooperated with the University of Oxford and others to develop a new miniature device, which can provide safe and high-definition medical imaging technology through the use of terahertz radiation to help doctors identify and treat deadly cancers as early as possible. Can you tell us the advantages of this device? What other issues need to be resolved? How far is it from clinical application?**

Our development of semiconductor nanowire based THz detectors which are sensitive to polarization, phase and amplitude has the potential to make a significant impact in the field. Many of the detectors are amplitude sensitive but not phase sensitive. We have developed both phase and amplitude sensitive broad bandwidth THz detectors with Oxford colleagues as part of Ms. Kun Peng’s PhD project. With these detectors, you have to do at least two measurements to get the polarization information while rotating the detector by 90 degrees. This is time consuming and complex to do realignment etc. Our new device, allows us to measure both x-and y-polarization state due to orthogonal arrangement of nanowires. This work is a collaborative effort involving Oxford, Strathclyde and Australian National University (ANU). The compact nature of these detectors will allow us to be able to pack multiple detectors within the THz beam spot providing high resolution THz images. In order to use these detectors in biomedical imaging applications, arrays of detectors need to be fabricated with appropriate electronics which is the next step. Hopefully industry will be interested in these detectors.

**2. As a leading expert on III-V nanomaterials and devices, what do you think are the most significant scientific and technological challenges in this field? What will be the most promising emerging or future technologies based on the III-Vs?**

III-V semiconductor based devices are widely used in electronics, optoelectronics and communication systems, CD/DVD players, LED lighting, high efficiency solar cells etc. In terms of the future, opportunities are there to develop single photon sources and detectors for quantum communications applications, flexible electronic and optoelectronic devices including flexible solar cells, nonlinear optics and meta-optical systems, nanolasers and detectors for miniaturized optical systems for holography, augmented reality, LiFi, LIDAR etc.

**3. You have been engaged for years in the research of III-V compound semiconductor materials and devices, and have made many achievements and won many important international academic awards, including the IEEE Third Millennium Medal in 2000, the Quantum Device Award in 2010, Electronics and Photonics Division Award of the Electrochemical Society in 2012 and UNESCO Medal in 2018. Would you like to tell us the key to good scientific research according to your own experience?**

The important thing is to choose something you are passionate about. Once you are enjoying what you are doing, good things happen. Choosing difficult scientific problems of global interest is important. Once we solve the problem, it will have a global impact. Good students and post-docs are critical to any research. I have been fortunate that I had many wonderful students and post-docs during my past 30+ years at the ANU and many of them are from China. Collaborations are also absolutely critical. We cannot be expert in everything. By focusing on our expertise and by collaborating with other experts who can bring complementary expertise can allow us solve scientific problems in a multi-pronged approach. I have also had wonderful collaborators from 30+ countries during my career including many from China as I first visited China in 1996 and I have seen phenomenal developments in science and technology and the society during the past 25+ years in China. Good team makes great science happen. My job is to inspire them, motivate them and recognize their contributions.

**4. You have been elected fellow of 12 professional societies and the scientific communities, for instance the Institute of Electrical and Electronics Engineers (IEEE), the American Physical Society (APS), the Materials Research Society (MRS), and the Optical Society (OSA). You have been involved in the work and activities, and have made great contributions. I wonder if you could tell us the benefits of these experiences to your scientific research career**.

Being involved in professional societies has allowed me to be able to work with colleagues from various parts of the world in promoting science and technology, mentoring young scientists and serving the community. I found many of my collaborators through interactions in the professional societies. Many of the friends I made through being involved in serving professional societies have been great supporters of our research. The pleasure of giving is much more immense than receiving. I have enjoyed giving something back to the community by being involved in various roles in professional societies. Professional societies bring people together to exchange ideas, knowledge and develop networks and nurture the discipline and people working in the field.

**5. You have initiated a fund to help Indian students to study in Australia. What prompted you to set up this fund? How does it work? How many students have been supported so far?**

First of all, I want you to know that the fund is open to students and young scientists from developing countries, not just from India, though many of the students come from India. Whenever we get requests from students from Europe to visit us for 3-6 months, we are happy to host them as they come with their financial support. However, whenever we get requests from developing countries, we say no to them as they need financial support and we do not have funding to support them. China is an exception as it has lots of programs to support students to visit international laboratories through scholarships such as the Visiting Student Scholarship by China Scholarships Council and also Chinese Academy of Sciences (CAS) and other universities have funding to support these visits. I have been feeling guilty as having started my life in a small village in India, without others giving me the opportunity I wouldn’t be where I am today. Two of my high school teachers and my parents have supported me to complete my high school and further studies. My wife Vidya and I have decided to create an Endowment at the ANU to support students and young scientists from developing countries. ANU has kindly agreed to provide matching funding to support our donations. The fund was launched in 2015 with our own donations and ANU’s support. We started by giving 4 scholarships to Bachelor degree or Master degree students to visit ANU to spend their summer (10-12 weeks) to gain research experience, showcase their abilities and develop their networks. We also give 1 young scientist fellowship per year. So far students and young scientists have come from India, Indonesia and Malaysia. Based on the success of our Endowment, ANU also started future research talents program to support students from India and Indonesia to visit ANU and work with my colleagues in various parts of the University. Our aim is to create an Endowment of AU$ 1 Million so that this will support up to 6 students and 1 young scientist every year in perpetuity. Every year we donate some funds to ANU and ANU matches our donation. Again this is all part of giving something back to the community and supporting the younger generation.

**6. As a distinguished scientist of the CAS President’s International Fellowship Initiative (PIFI), you have visited Changchun Institute of Optics, Fine Mechanics and Physics (CIOMP) and later received a visiting scholar from CIOMP to work with your team in ANU. What is your impression of CIOMP?**

I have enjoyed my visit to CIOMP. I was very impressed with CIOMP and its research activities. The scales of optics activities are very impressive. From nanoscale optics to huge optics for telescopes and other applications, it is truly admirable. The ability of CIOMP to cover from fundamental science and industry applications to creating industries is excellent and I wish continued success to CIOMP in its endeavors.

**7. You served as an editor of** ***Light: Science & Applications*** **(Light) until the beginning of 2020. Could you please talk about your reasons for joining and quitting?**

It was a pleasure and honor for me to serve as an Editor of Light and I enjoyed working with the Light team. In January 2020 I took up the role of Editor-in-Chief of Applied Physics Reviews. My contract for this position will not allow me to be an Editor of other journals. In view of this, I had to step down from my role as an Editor of Light. I wish continued success to Light.

**8. Do you have any advice for young researchers about how to do research well?**

The most important thing in life is self-belief and developing a can-do, positive attitude. Choosing something they are passionate about and working hard and smart to achieve their goals. Perseverance and persistence are important in life. We fail many times, but determination to succeed will allow one to keep going and to get over the failures. Be kind and generous to others and develop good communication skills (both oral and written) and ability to work as part of a team is important in life. Work-life balance is important and also one needs to look after their health. Health is the most important thing in life, but we ignore it and only realize its importance once we lose it. Everything is possible in life. One sets a goal, works smart and hard and persists with the goal despite the hurdles. Sky is the limit! Keep trying and never give up! Good luck!

**Figure 1:**
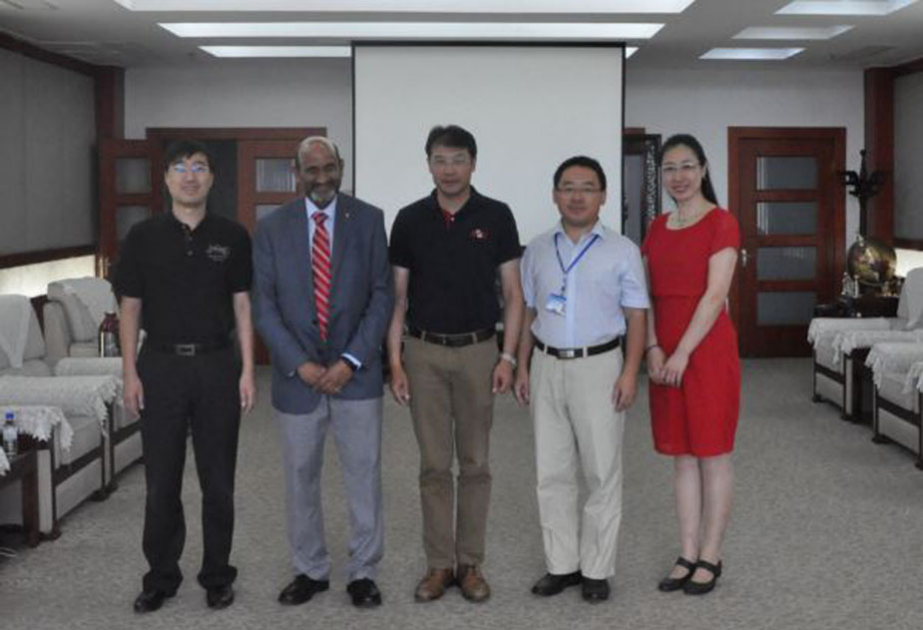
Fig. 1 Prof. Jagadish visited CIOMP as a CAS PIFI Distinguish Fellow in 2016.

**Figure 2:**
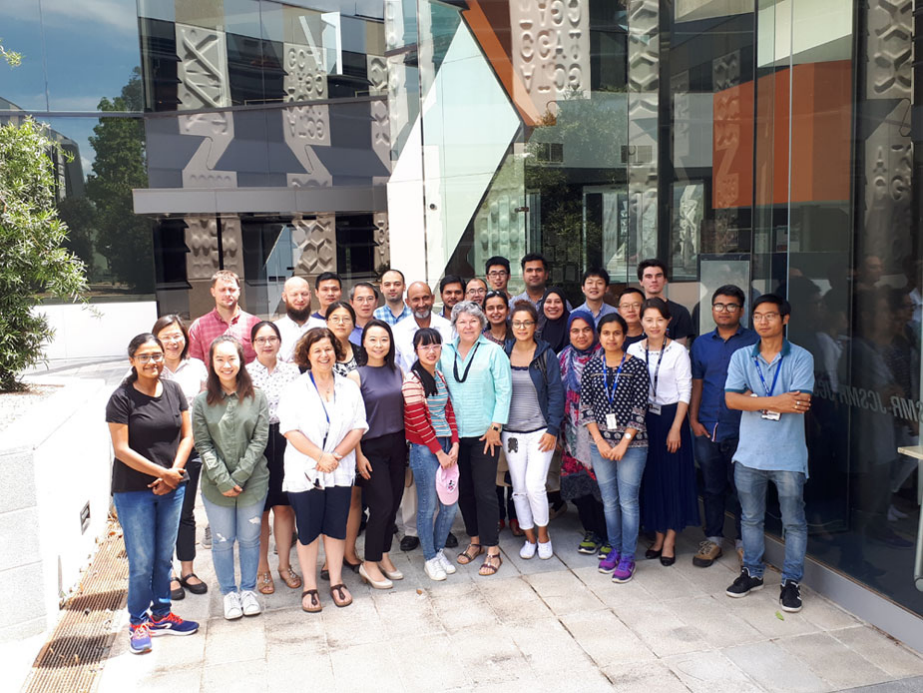
Fig. 2 Prof. Jagadish’s group with Dr. Lijie Wang from CIOMP as a Visiting Scholar in ANU in 2019.

**Figure 3:**
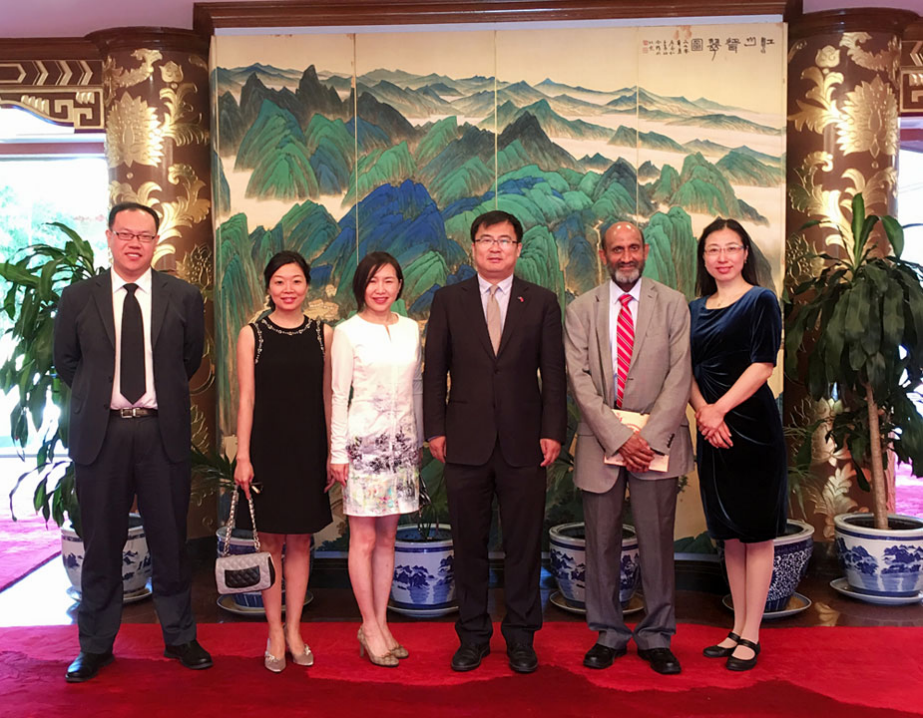
Fig. 3 The Science and Technology Counselor Mr. Qiang Wang (third from right) of the Chinese Embassy in Australia met with Prof. Jagadish and two founding members of Light.

**Figure 4:**
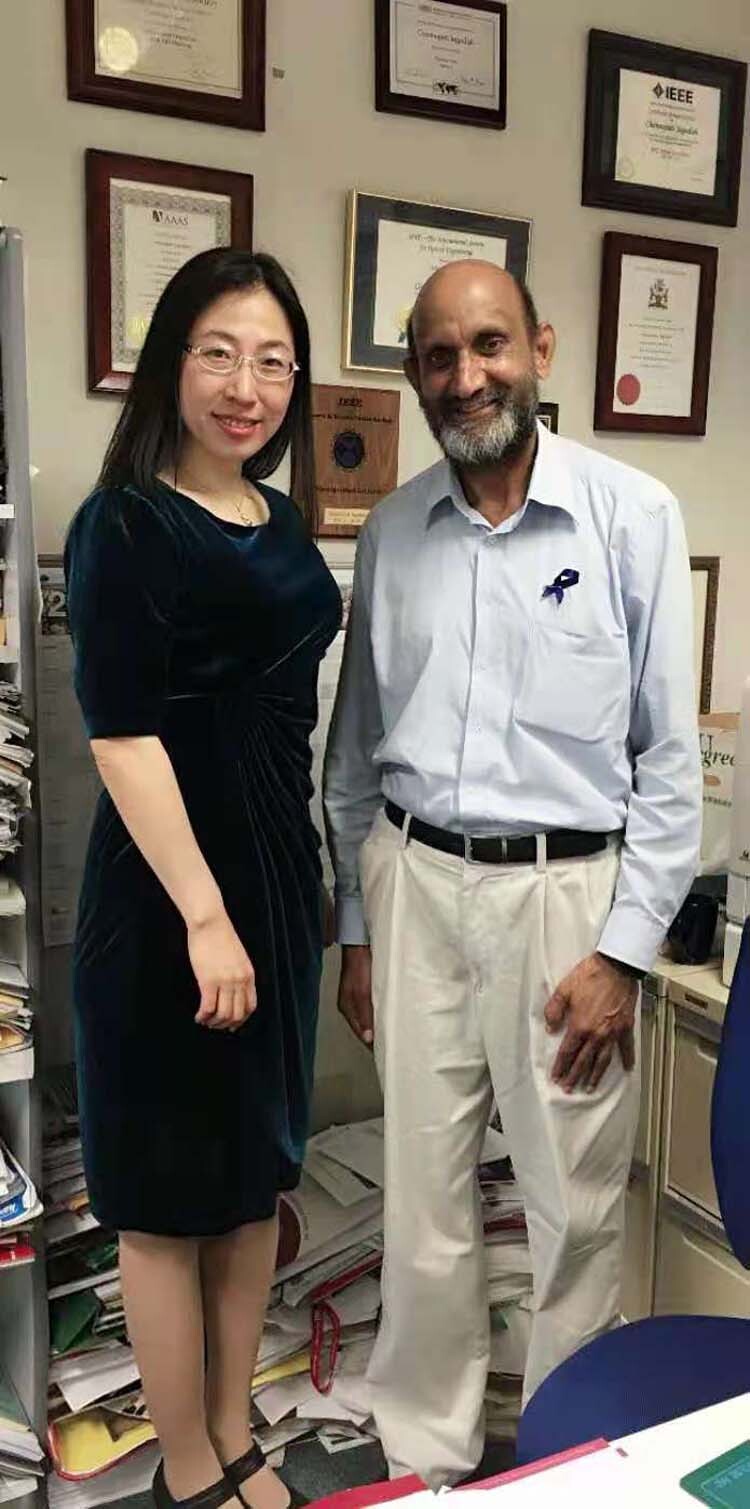
Fig. 4 Prof. Jagadish with Ms. Hui Wang, Light special correspondent for this interview, in his office when Ms. Wang visited his laboratory in 2016.

**Light special correspondent**

*Hui Wang is the Deputy Director of the Office of International Cooperation in the Changchun Institute of Optics, Fine Mechanics and Physics (CIOMP), Chinese Academy of Sciences (CAS). She currently works on international communication and cooperation for the CIOMP and was a founding member for the Nature Publishing Group and CIOMP joint journal Light: Science & Applications. She is the founder of “Rose in Science” and has published several articles in Acta Editologica, Inter-national Talent, etc., and was invited to take an interview by SPIE Women in Optics, which was published in 2015*.

